# OTU Analysis Using Metagenomic Shotgun Sequencing Data

**DOI:** 10.1371/journal.pone.0049785

**Published:** 2012-11-26

**Authors:** Xiaolin Hao, Ting Chen

**Affiliations:** Molecular and Computational Biology, University of Southern California, Los Angeles, California, United States of America; University of Waterloo, Canada

## Abstract

Because of technological limitations, the primer and amplification biases in targeted sequencing of 16S rRNA genes have veiled the true microbial diversity underlying environmental samples. However, the protocol of metagenomic shotgun sequencing provides 16S rRNA gene fragment data with natural immunity against the biases raised during priming and thus the potential of uncovering the true structure of microbial community by giving more accurate predictions of operational taxonomic units (OTUs). Nonetheless, the lack of statistically rigorous comparison between 16S rRNA gene fragments and other data types makes it difficult to interpret previously reported results using 16S rRNA gene fragments. Therefore, in the present work, we established a standard analysis pipeline that would help confirm if the differences in the data are true or are just due to potential technical bias. This pipeline is built by using simulated data to find optimal mapping and OTU prediction methods. The comparison between simulated datasets revealed a relationship between 16S rRNA gene fragments and full-length 16S rRNA sequences that a 16S rRNA gene fragment having a length >150 bp provides the same accuracy as a full-length 16S rRNA sequence using our proposed pipeline, which could serve as a good starting point for experimental design and making the comparison between 16S rRNA gene fragment-based and targeted 16S rRNA sequencing-based surveys possible.

## Introduction

Metagenomics is a new area of study which has undergone rapid developement as our ability to sequence microorganisms in given environmental samples without culturing them got improved. Consequently, research in this area has provided important insights into a variety of topics, ranging from the relationship between bacteria and human health [1], [2] to biogeochemical activities that occur in the ocean [3]. Alongside the development of sequencing technology, metagenomic studies have also benefited from increased data size that allows the generation of billions of metagenomic shotgun sequences [4] or targeted 16S rRNA gene sequences directly from an environmental sample [5]. However, this explosion of sequencing data has not yielded greater insight into microbial diversity, as previously provided by surveys on a smaller scale, mainly because of errors and bias in the data. Specifically, artifacts that arise from PCR and sequencing errors [3], [6] might result in overestimation of microbial diversity. Moreover, the results of targeted 16S rRNA gene sequencing data surveys are also affected by potential primer bias [7] based on the use of “universal primers”. Computational methods have been developed to address errors in PCR/sequencing [3], [8], [9], but primer bias issue remains largely untouched.

On the other hand, metagenomic shotgun sequencing data is naturally immune to primer bias. Global Ocean Survey (GOS) is one of the first and still on-going large-scale research projects using this type of data [10]. However, the limited availability of known bacterial genomes makes it difficult to associate sequencing reads with operational taxonomic units (OTUs), and the highly dynamic structure of the underlying bacterial genomes [11] introduces extra complexity to the data analysis. Fortunately, the data explosion has also made available another new data type, 16S rRNA gene fragments, which may leverage the advantage of both 16S rRNA genes and metagenomic shotgun sequencing data. Previous surveys have shown that targeted 16S rRNA gene sequencing data tended to overestimate or underestimate the abundance of some bacteria phyla when compared with 16S rRNA gene fragments from the metagenome of the same community [2]. This difference is potentially caused by the primer bias of targeted sequencing, thus lending support to the use of 16S rRNA gene fragments from metagenomes. Some researchers have developed computational methods for analyzing the mean species diversity at the habitat level (also known as α-diversity) and the differentiation among habitats (also known as β-diversity) using this type of data [12], [13], while others have taken advantage of the current public 16S rRNA gene databases [14], [15], [16] to identify OTUs through database search. Despite the increasingly popular metagenomic analysis using this new type of data, lack of statistically rigorous comparison between 16S rRNA gene fragments and other data types makes it difficult to interpret previously reported results using 16S rRNA gene fragments. For example, it is possible that the observed difference in the results of the two data types can be attributed to the different methods used for analyzing targeted 16S rRNA gene sequencing data and 16S rRNA gene fragments. Thus, it is of great interest to investigate 16S rRNA gene fragments and establish a standard analysis pipeline such that its comparison with traditional 16S rRNA gene-based surveys will yield the “true” differences in the data.

Therefore, in this article, we focus on establishing such an analysis pipeline by conducting data analysis through 16S rRNA gene databases using simulated data. Since individual variable/hyper-variable regions of 16S rRNA gene have inconsistent and more complicated features [17] that introduce further bias to OTU abundance estimation, which is out of the scope of this paper, we focus on almost-full-length sequences for targeted-sequencing. The work can be described in two steps:

We first generated simulated 16S rRNA gene fragments datasets with various read length and read numbers from a set of known full-length 16S rRNA gene sequences, thus the underlying taxonomic profile (the “ground truth”) of the generated datasets are known. Then we mapped these simulated 16S rRNA fragments to annotated full-length 16S rRNA sequence databases using BLAST, and used the BLAST mapping results to generate predicted taxonomic profile for each dataset. By tuning above mapping procedure and testing different type of methods of generating OTU predictions using the mapping results, we decided that nearest-neighbor approach (see Methods) is the optimal approach in terms of three performance metrics: sensitivity, specificity and Bray-Curtis dissimilarity.

The second step is to establish a relationship between 16S rRNA gene fragments and full-length 16S rRNA sequences. To be more specific, we want to answer the question “How many 16S rRNA gene fragments with average length *L* are able to generate taxonomic profile prediction consistent with that of *Y* almost full-length 16S rRNA sequences when there is no bias in the data?” To answer this question, we first generated a set of simulated full-length 16S rRNA sequence datasets with various read numbers by sub-sampling from the set of full-length sequences we used to generate simulated 16S rRNA gene fragments datasets above. Then taxonomic profile predictions were generated for both simulated 16S rRNA gene fragments datasets and simulated full-length datasets using the previously-determined optimal approach. By comparing the sensitivity, specificity and Bray-Curtis dissimilarity achieved by these datasets using Mann-Whitney U-test (see Methods), we are able to draw conclusion that 16S rRNA gene fragments, with an average read length of at least 150 bp, could provide the same level of resolution as full-length 16S rRNA gene sequences in terms of α-diversity, Bray-Curtis dissimilarity, sensitivity and specificity.

Our established analysis pipeline that can test for “true difference” between a 16S rRNA gene fragment dataset and a targeted-sequencing dataset sharing the same underlying microbial community uses above conclusion reversely: First, we check if the 16S rRNA gene fragments are >150 bp in length. Then we perform necessary sub-sampling of the two datasets to generate similar-sized sub-datasets. Finally we process these sub-datasets using aforementioned optimal approach and make comparison between them using a likelihood ratio test (see Methods). These sub-datasets, according to our analysis, should generate OTU abundance estimations that are statistically similar to each other. Thus if statistically significant difference are detected, it can be concluded that there are bias in the data used.

We used this pipeline to analyze a set of gut data taken from the Human Microbiome Project [2]. These data included metagenomic shotgun sequences, full-length 16S rRNA gene sequences and sequences from V2 and V6 hypervariable regions of 16S rRNA gene. We confirmed the statistically significant difference between the OTU abundance levels estimated from 16S rRNA gene fragments and those estimated from other data types at various taxonomic levels, suggesting potential bias in this data. We also showed that 16S rRNA gene fragments gave high-level predictions consistent with previous studies. Though GOS also provides a good data source, considering its relatively low data coverage (∼100 16S fragments per sample), we didn’t use it for validation purposes.

The C++ program for the OTU analysis using metagenomic shotgun sequencing data proposed in this study is available at https://code.google.com/p/shotgun-metagenomics-analysis-framework/.

## Materials and Methods

### 1.1. Data

Two data types were used in the study: simulated data and real data.

Two groups of simulated data, as shown in Table 1, were used in the study. The first group of datasets consisted of a total of 540 simulated 16S rRNA gene fragment datasets with various read lengths (50–300 bp) and sizes (100–10000 sequences) uniformly generated by random sampling from 9,773 unique nearly full-length 16S rRNA gene sequences [2]. The sampling procedure is done by first choosing a full-length sequence, then randomly choosing a position on the full-length sequence where the distance between the position and the end of the sequence is larger than the current sampling read length. Then this position is used as the starting point of the next read. For each specific choice of read length and data size, 10 simulated datasets were generated in order to study the variation of results. The choice of these parameters reflected current technology in metagenomic sequencing. As such, the range of read length covered 50 bp to 150 bp from the Illumina sequencing platform to 200 bp or longer from the 454 sequencing platform. Although we tested other read lengths between the numbers chosen, the results did not significantly improve. The dataset size followed an assumption that the average coverage was less than 0.1×. The second group of datasets consisted of 5 simulations of full-length 16S rRNA gene sequence datasets, each with a different data size and 10 replicates. Each replicate was generated by the random sampling from the original 9773 sequences. The sizes of these datasets were designed to provide comparable coverage (<0.1×) with the simulated 16S rRNA gene fragment datasets.

**Table 1:** Summary of Shotgun and Full-length Simulated Datasets.

	16S rRNA Gene Fragments	Full-length 16S rRNA Sequences
Read Length (bp)	50,65,85,100,150,200	∼1200–1600
Data Size	100, 200, 400, 800, 1000,2000,4000,8000,10000	100,200,400,800,1000
Total Datasets (with 10 replicates)	6×9×10 = 540	1×5×10 = 50

The real data consisted of nearly full-length 16S rRNA sequences, V2 amplicon of 16S rRNA gene, V6 amplicon of 16S rRNA gene, and 454 whole genome shotgun sequencing data introduced by Turnbaugh et al. [2]. This study used the following 18 samples, all having four data types: TS1, TS2, TS3, TS4, TS5, TS6, TS7, TS8, TS9, TS19, TS20, TS21, TS28, TS29, TS30, TS49, TS50 and TS51.

### 1.2. Mapping

#### 1.2.1. Databases

Two databases were used to map metagenomic shotgun sequencing data and extract taxonomic information. The first dataset, referred to as “raw database”, consisted of 1428381 high-quality 16S rRNA gene sequences annotated in RDP. The second dataset, referred to as “redundancy-reduced database”, was built by clustering the 1428381 sequences at 3% distance threshold using a program called *uclust* [18] (version 1.1.579 for 64-bit Linux platform) with default parameters. The final size of the redundancy-reduced database is 252314. The two datasets were then built into BLAST databases. Sequences were shuffled before construction of the databases since they were originally grouped sequentially by their RDP taxonomy annotation. The 9773 original full-length sequences were removed to avoid self-hitting.

#### 1.2.2. Mapping shotgun sequencing data to databases by BLAST

BLASTN was used to map the data to the databases. We used very stringent E-value and similarity cut-offs, as shown in Table 2, so that boundary reads across the 16S gene and other parts of bacteria genomes could be excluded.

**Table 2:** E-value and Identity Cut-offs.

Read Length (bp)	E-value cut-offs	Similarity cut-offs
50	1e–10	90
65	1e–13	90
85	1e–17	90
100	1e–20	90
150	1e–30	90
200	1e–40	90
Full-Length	1e–200	90

**Table 3:** Comparison between the Complete Database and the Compressed Database.

	Complete Database	Compressed Database
Bray-Curtis Dissimilarity	0.1205142±0.064813	0.1213089±0.065371
Sensitivity	0.620783±0.203592	0.604533±0.227941
Specificity	0.877164±0.019811	0.879328±0.020517
Mapping Speed (Base/Sec)	55	6

### 1.3. OTU Prediction

By mapping metagenomic shotgun sequencing data to a large 16S gene database, we are able to extract out reads which are potential 16S rRNA gene fragments. However, a significant portion of the 16S rRNA gene fragments will get multiple BLAST hits in closely related taxonomic groups, even under very stringent criteria, i.e., similarity cut-off >99% and mapping length >95%, based on the presence of highly conserved regions in 16S rRNA genes, which poses difficulty for accurate OTU abundance estimation. Methods have been proposed to handle multiple hits. One popular approach, which is used in MEGAN [19], assigns a read to the Lowest Common Ancestor (LCA) of its mapped taxonomic groups. In practice, however, this approach fails to assign most 16S rRNA fragments to the genus-level taxonomic group. Thus, we propose the following four approaches where we only consider those BLAST hits with >90% of read length mapped and >95% similarity for the genus-level OTU analysis.

#### 1) Nearest Neighbor (NN)

Each taxonomic group consists of multiple 16S rRNA gene sequences to which a read may have multiple hits. The nearest neighbor approach assigns each read to the taxonomic group with the highest BLAST score, or, if two or more groups have the same score, to all of those with equal weights summing up to 1. The abundance of a taxonomic group is the number of the weights of its reads.

This straightforward method could have many different variations by giving different definitions for “nearest neighbor”. For example, we may consider a read belongs to a taxonomic group if its BLAST hit score in this group is some margin better than hit scores in any other groups. However, preliminary results showed that using above mentioned method, on average >90% of the reads can be assigned correctly, and there is no way to correctly assign the rest 10% by simply looking at their sequence content since they are 100% identical to many taxonomic groups at even phylum-level. Thus we used above approach as a representative of the nearest neighbor approach family.

**Figure 1:**
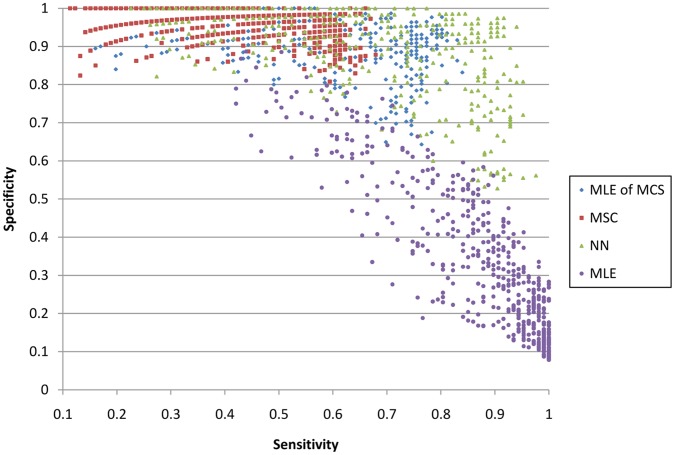
Comparison of four computational methods for integrating taxonomic information. MLE stands for the maximum likelihood estimation method, MSC stands for the Minimum Set Covering method, MLE of MCS stands for the MSC method followed by the MLE method, and NN stands for the nearest neighbor method. Each data point indicates the sensitivity and specificity achieved by one simulated dataset using corresponding method.

**Figure 2:**
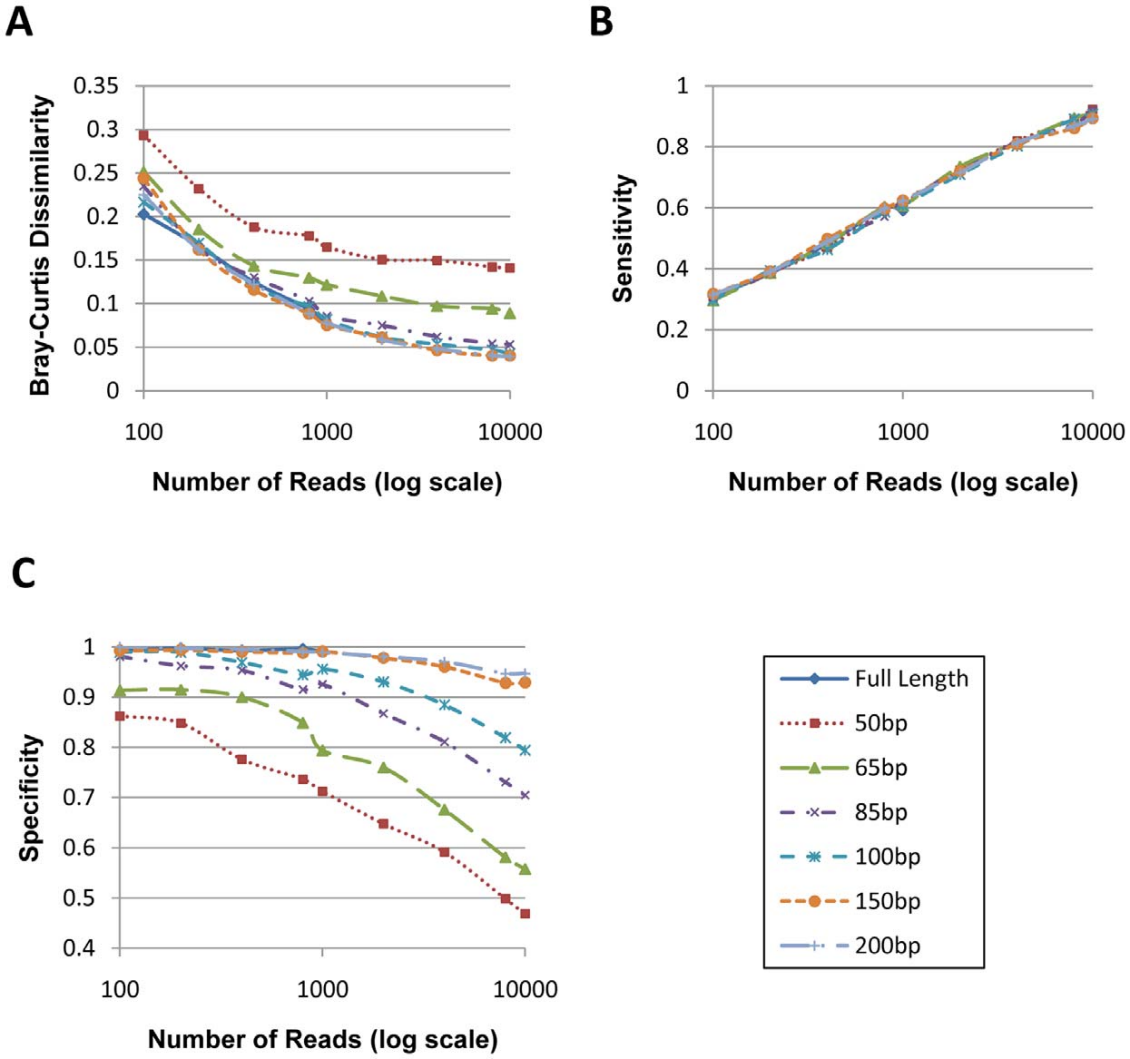
OTU prediction performance evaluation for all simulated datasets. X-axis indicates read number, y-axis indicates value of corresponding performance metric ((A) for Bray-Curtis dissimilarity, (B) for sensitivity and (C) for specificity), each colored line indicates a different read-length.

#### 2) Maximum Likelihood Estimation (MLE)

Since probabilistic approach gives chance for a read to be assigned to any taxonomic group it gets a BLAST hit in, these approaches are expected to generate results with higher sensitivity. Thus we tested an MLE method for OTU prediction. The setting we used is a basic EM algorithm for inferring component abundance of a mixture model.

The likelihood model is described as follows: Assume that read *i*, 1≤ *i* ≤ *n*, has *k* BLAST-hits in taxonomic group *j*, 1≤ *j* ≤ *m*. For the *m*-th hit, let *l_m_* be the length of the local alignment reported by the BLAST, and let *s_m_* be the percentage of identities in the local alignment. Then the likelihood that read *i* is generated from the taxonomic group *j* is
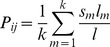
(1)where *l* is the length of the reference full-length 16S rRNA gene sequence.

Let *π_j_* be the abundance of a taxonomic group *j* in a sample, and let *Z_i_ = j* be the latent variable indicating that read *i* is assigned to a specific taxonomic group *j*. Thus for all the *N* reads and *M* taxonomic groups, the full likelihood function is then:
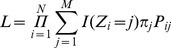
(2)


A log transformation gives:
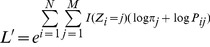
(3)

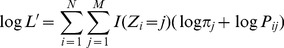
(4)


In order to get the MLE of {*π_j_*}, we impute {*I(Z_i_ = j)*} in the above formula with its conditional expectation:
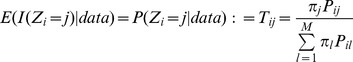
(5)


A simple EM algorithm [20] is used to determine the maximum likelihood estimate of the abundance of a mixture. For the *t*-th iteration,

E-step:
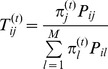
(6)


M-step:
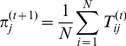
(7)


For most reads in our study, the reported BLAST hits in different taxonomic groups were not mathematically different in terms of such parameters as alignment length or percentage of identities. Therefore, using a different likelihood model, or further fine tuning the above parameters, would not provide any significant difference in result.

#### 3) A Greedy Algorithm for the Minimum Set Covering (MSC) Problem

The third approach belongs to the category of maximum parsimony. Since this type of approach put more emphasis on high-abundance OTUs, the results are expected to be high in specificity.

If we define taxonomic groups (such as different genera) as “sets”, *s*_1_, *s*_2_,*…*, and reads as “elements”, *r*_1_, *r*_2_,*…*, then *r_i_*∈*s_j_*, or read *r_i_* is covered by group *s_j_*, if read *r_i_* gets a BLAST hit in the taxonomic group *s_j_*. Using such definition, the objective of the maximum parsimony becomes finding the smallest number of taxonomic groups that can cover all the reads, which is exactly the same as the minimum set covering problem, which is known to be NP-complete [21]. Since a polynomial-time solution does not exist, we use a greedy algorithm to determine a covering set. The greedy algorithm first selects a set covering the maximum number of uncovered elements and then repeatedly adds a new set which can cover the maximum number of uncovered elements until every element is covered by the selected sets. Finally, we select a set of taxonomic groups (sets) that explains all sequenced reads.

To estimate the abundance of the selected taxonomic groups, we weight each read by the following rule: if a read hits *n* selected groups, it carries 1/*n* weight in each group. Thus the abundance of a taxonomic group is estimated as the total weights of its reads.

**Figure 3:**
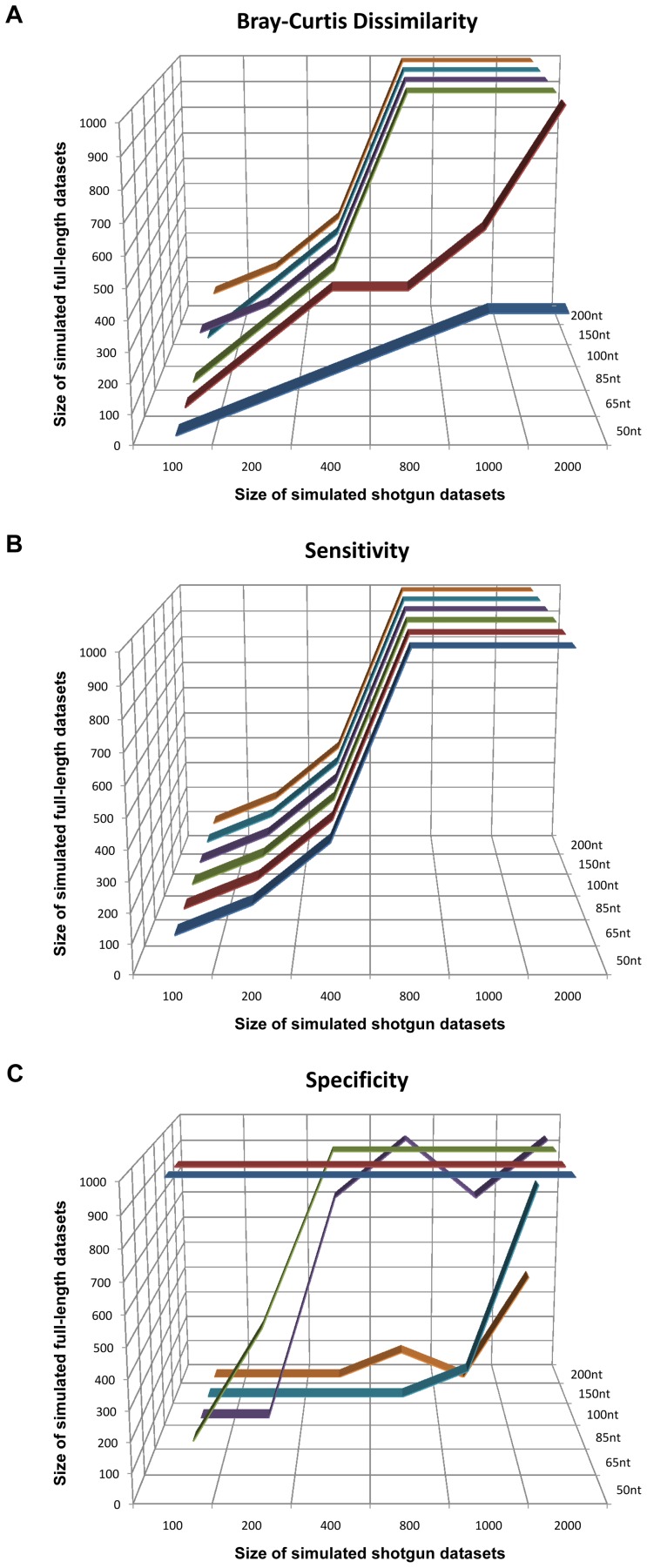
A plot showing relationship between 16S rRNA fragments and full-length 16S rRNA sequences. i.e.: A data point in subplot (A) at (500, 400, 65 nt) indicates that 500 16S rRNA gene fragments with average read length 65 nt can achieve similar Bray-Curtis dissimilarity as 400 full-length 16S rRNA gene sequences. Subplots are based on Bray-Curtis dissimilarity (A), Sensitivity (B) and Specificity (C).

#### 4) Maximum Likelihood Estimate of Minimum Covering Set (MLE of MCS)

This approach is similar to the aforementioned greedy algorithm, except that we apply an EM algorithm to estimate the abundance of the selected taxonomic groups to see if this approach could take advantage of both MLE and MCS to generate results with both high sensitivity and specificity.

### 1.4. Performance Evaluation Metrics of OTU Prediction Result

Based on their taxonomy in RDP, we consider the abundance estimation of the original 9773 nearly full-length 16S rRNA gene sequences to be the ground truth. Several metrics, including Bray-Curtis dissimilarity, sensitivity and specificity, are used to compare the ground truth with OTU prediction results of each simulated dataset. Here, sensitivity is defined as the percentage of the taxonomic groups in the ground truth found by analyzing the simulated data. Specificity is defined as the percentage of the taxonomic groups estimated from the simulated data which are also in the ground truth.

### 1.5. Using Mann-Whitney U-test to Compare Simulated Datasets

We want to answer the question “How many 16S rRNA gene fragments with average length *k*_1_ are needed to generate taxonomic profile prediction consistent with that of *Y* almost full-length 16S rRNA sequences when there is no bias in the data?” To address this question, we use the approach described below.

First, we denote 1) the simulated 16S rRNA gene fragment dataset with read length *k*_1_ and read number *n*_1_ as dataset (*k*_1_, *n*_1_) and 2) the simulated full-length 16S rRNA gene sequence dataset with read number *n*_2_ as dataset (*full*, *n*_2_) below. Since each such defined dataset has 10 replicates, we can use the Mann-Whitney U-test [22] to test the difference between each simulated 16S rRNA gene fragment dataset and each full-length 16S rRNA gene sequence dataset measured by the Bray-Curtis dissimilarity, sensitivity and specificity, using 5% significance level We define two datasets, i.e., datasets (*k*_1_, *n*_1_) and dataset (*full*, *n*_2_) to be equivalent if (1) there is no significant difference between their performance measures, in terms of sensitivity, specificity, or the Bray-Curtis dissimilarity, or (2) dataset (*k*_1_, *n*_1_) gives better performance than dataset (*full*, *n*_2_), but worse performance than dataset (*full*, *n*_3_) where *n*_3_>*n*_2_.

**Figure 4:**
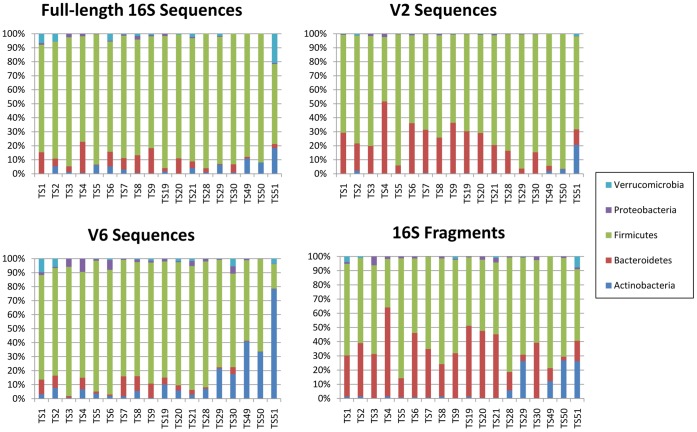
Abundance estimations of major phyla for all four data types in a human gut microbiome.

### 1.6. Comparison of Real Datasets

#### 1.6.1. Likelihood Ratio Test (LRT)

Since for real data, we usually have only one replicate for each sample, thus we are unable to compare them using Mann-Whitney U-test. Also, current real 16S rRNA gene fragment data are usually of limited size, which raise another concern that the observed difference between the genus-level abundance estimation of 16S rRNA gene fragments and that of full-length 16S rRNA gene sequences maybe simply by chance under low coverage. We use a Likelihood Ratio Test (LRT) to address above concern and determine if the observed difference between the genus-level abundance estimation of 16S rRNA gene fragments and that of full-length 16S rRNA gene sequences is from low coverage (null hypothesis H_0_) or some other biases (H_1_). If we assume that each dataset is a sample from a multinomial distribution *Mult(n*, *p*_1_, *p*_2_, *p_k_*…*)*, the maximum likelihood estimates *p′*_1_, *p′*_2_, …, *p′_k_* of the cell probabilities *p*_1_, *p*_2_, …, *p_k_* are then the corresponding genus-level abundance estimation. Under the null hypothesis, this abundance estimation is determined by estimating the abundance of different genera after pooling two datasets together. The test statistic is the log ratio of the likelihood under the null and the alternative hypotheses:
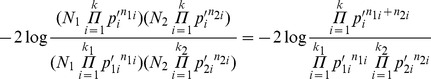
(8)where *N*_1_ and *N*_2_ are the numbers of the 16S rRNA gene fragments and the full-length 16S rRNA sequences for this sample, respectively. *k*_1_ and *k*_2_ are the numbers of detected genera, *p′*_1*i*_ and *p′*_2*i*_ the estimated abundances of the *i*-th detected genera, and *n*_1*i*_ and *n*_2*i*_ the numbers of sequences assigned to the *i*-th genus, respectively. *p′_i_* is the pooled estimate of the abundance of the *i*-th genus. *k* is the number of genera detected by at least one sample.

This test statistic follows a chi-square distribution with the degree of freedom equal to *k*_1_+*k*_2_−*k*+1 asymptotically. A *p*-value is thus calculated correspondingly. If we assume that the two samples are drawn from the same underlying multinomial distribution and there is no other factor affecting the sampling process, then the test will not reject the null hypothesis, even if the coverage is low (small *N*_1_ and *N*_2_).

Since the gut microbiome data we used have 18 samples to be tested simultaneously, the *p*-value is then corrected using the Benjamini and Hochberg False Discovery Rate (FDR) [23]. Although the following analysis is for the genus-level only, the test can be applied to any taxonomic level by simply using the abundance estimation at the desired taxonomic level.

**Figure 5:**
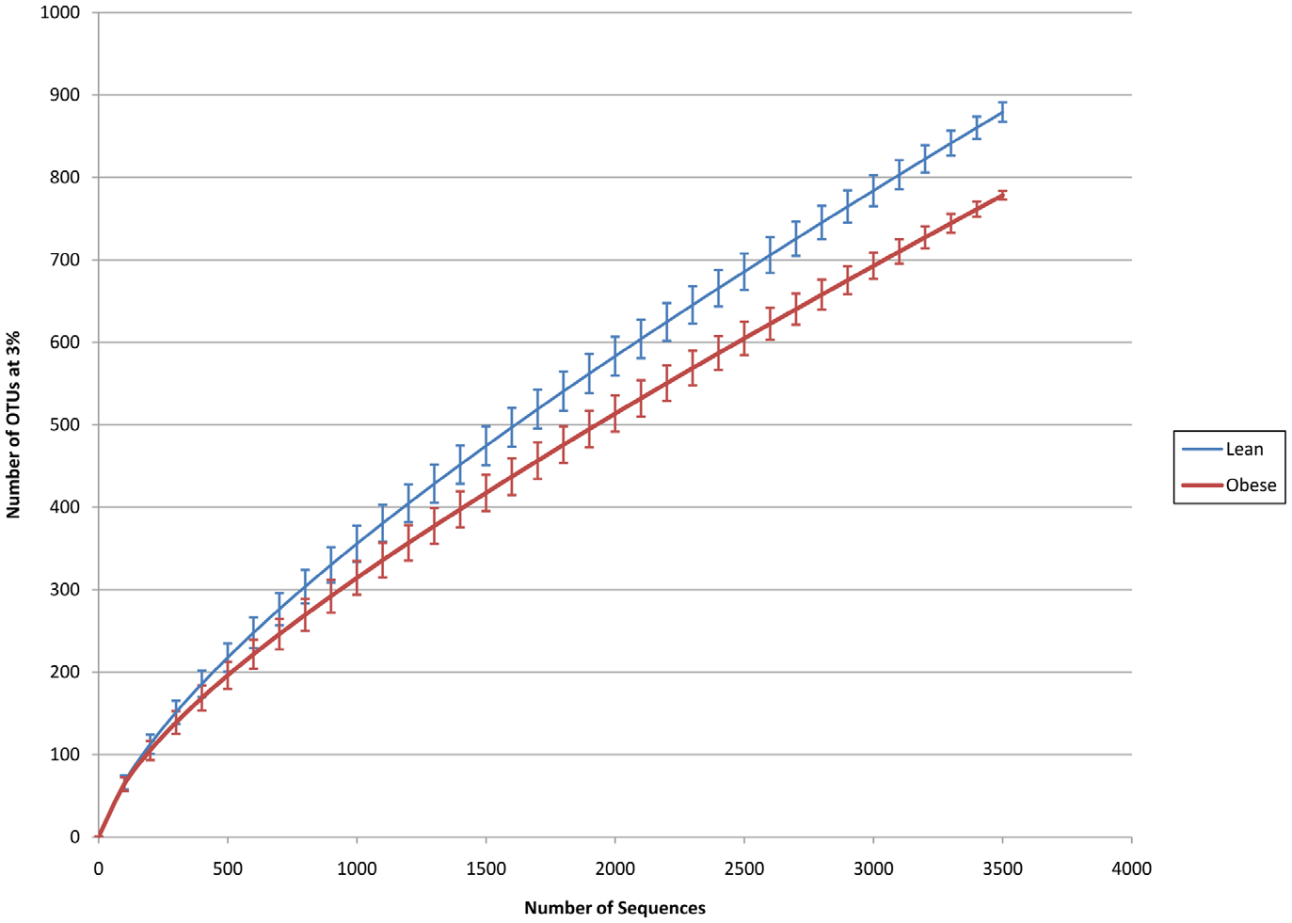
Rarefaction curve of lean and obese samples. Error bars indicating 95% confidence interval.

#### 1.6.2. Wilcoxon signed-rank test [24]

The Wilcoxon signed-rank test is used to find the genera whose abundance estimation is significantly different between two different data types. The abundance estimation of a certain genus for a data type forms an 18-dimensional vector representing the estimated abundance levels from the 18 samples. The Wilcoxon signed-rank test is then performed on each pair of such vectors with a significance level of 0.05.

#### 1.6.3. Diversity analysis

Mothur [25] software (version 1.22.2) with default parameter settings is used for diversity analysis. First, the RDP-aligned best hit full-length 16S sequences (determined as described above) for each 16S rRNA gene fragment is put into a fasta file, while information, which identifies the sequence from which the sample is generated, is stored in a separate “group” file. These 2 files are then imported into mothur for downstream analysis, including the hierarchical clustering and the rarefaction curve calculation.

### 1.7. Program Availability

All statistical tests, performance metrics calculation and analysis procedures are implemented in C++, which is available at https://code.google.com/p/shotgun-metagenomics-analysis-framework/.

## Results

### 2.1. Test for Optimal Mapping Database

We compared the mapping results using the raw database with those using the redundancy-reduced database (see Methods). As shown in Table 3, we measured the mapping speed by how many bases could be mapped per second on a single Opteron 885, 2.6 GHz CPU when using two different databases. Given the results, we would recommend using the redundancy-reduced database since the mapping process is at least several orders of magnitude faster than using the raw database, while the performance, in terms of Bray-Curtis dissimilarity, sensitivity and specificity, is not significantly different (two-sided *t*-test, p-value = 0.834, 0.188, 0.066 for three metrics, respectively).

### 2.2. Test for Optimal OTU Prediction Method

On average, 75.2% simulated reads get BLAST hits with highest scores in at least 2 different taxonomic groups, suggesting necessity of exploring different approaches to integrate this information.

Figure 1 compares the performance of the four computational methods. Each colored point indicates the sensitivity and specificity achieved for applying a specific method into one of the 540 simulated shotgun datasets.

Overall, results suggest that the nearest neighbor (NN) method produces the most accurate results. The maximum likelihood estimate (MLE) method consistently has the lowest specificity, while the greedy algorithm (MSC) consistently has the lowest sensitivity. The MLE of MCS method has better sensitivity than the MSC method, but with lower specificity. The NN method achieves the best combination of sensitivity and specificity (the most upper-right corner of Figure 1). Also, in terms of Bray-Curtis dissimilarity, the NN method consistently achieves the best performance, which indicates the most accurate overall abundance estimation. Although we lose some specificity for datasets with short read length, the NN approach is the best choice based on the above results, using current sequencing platforms where read length usually ranges from 100 to 400. Thus, we choose this approach in our proposed framework.

### 2.3. Comparison of Simulated Data to Establish Relationship between 16S rRNA Gene Fragments and Full-length 16S rRNA sequences

On average, 99.5% of the simulated 16S rRNA gene fragments were mapped. For the 10 datasets generated from each combination of read length and the read number, we calculated the average Bray-Curtis dissimilarity, sensitivity and specificity based on the weighted-nearest-neighbor method. Results are shown in Figure 2.

The result in Figure 2 shows that the read number is the factor dominating sensitivity, while read length is negligible. Thus, by carefully choosing mapping criteria, reads as short as 50 bp can achieve sensitivity similar to the full-length sequences. As such, the Illumina platforms with their higher throughput will have the best sensitivity in detecting new taxonomic group. However, in terms of the Bray-Curtis dissimilarity and specificity, both read length and the read number play an important role. Together, they give a better sense about the accuracy of the abundance estimation. As expected, when read number is high, specificity is low, mainly because more reads will fall into highly conserved regions of 16S rRNA genes, and these reads are harder to be correctly assigned. In addition, when reads are longer, the data tend to have better immunity to this specificity-diminishing effect. This can be largely explained by the fact that longer reads are more likely to contain taxon-specific information and are thus less likely to be assigned incorrectly.

Although the above results show that the simulated 16S rRNA gene fragments can produce highly accurate abundance estimation, the conclusion only holds true for the community represented by the 9773 full-length 16S sequences from which we sampled the data. As such, both changes in community membership and structure could affect the above results. For example, based on the above results and given the limited genus-level diversity for this specific community, we conclude that a shotgun sample with an average read length of 200 bp and ∼300 reads mapped to a 16S database provides an abundance estimation equivalent to 1000 full-length 16S sequences generated from the same community. However, this is apparently not true if the community is more diverse, say, containing 500 genera. Thus, we used a comparative method to predict the abundance estimation accuracy when using the 16S rRNA gene fragments.

By comparing the results of the simulated 16S rRNA gene fragments and full-length samples, we are able to establish a relationship between them (Figure 3). This relationship was built by using pair-wise Mann-Whitney U-test to test the significance (*p*-value <0.05) of the difference between Bray-Curtis dissimilarity, sensitivity and specificity of full-length and shotgun samples (see Methods). From these results, we concluded that *n* 16S rRNA gene fragments would achieve the same level of accuracy as *n* full-length 16S rRNA sequences when read length is ≥150 bp. In other words, it is highly likely that a read from a 16S rRNA gene ≥150 bp in length would contain enough information to characterize the whole 16S rRNA gene. Under these conditions, either 454 or pair-end Illumina may be the best sequencing platforms since either can achieve a read length >150 bp and thus, according to our conclusion, *n* 16S rRNA fragments data from these two platforms would achieve the same level of accuracy as *n* full length 16S rRNA sequences.

### 2.4. Use the Proposed Pipeline to Compare Real Data for Potential Bias in Data Generation

By using our proposed analysis pipeline, we studied the human gut microbiome data introduced by Turnbaugh et al. [2]. There were 18 samples which had all four data types (WGS, full-length 16S, V2 region and V6 region) and were thus used for our comparative study. The phylum-level abundance estimations for all four data types are shown in Figure 4.

According to the result from simulated data shown in Figure 3, the shotgun data (>200 bp, 406±105 16S rRNA gene fragments) should have a level of accuracy similar to the full-length data (314±31 full-length 16S rRNA sequences). However, the Bray-Curtis dissimilarity between the two data is highly significant, using the two-sided *t*-test with p-value <10^−5^. This could result from potential technical bias in 16S rRNA gene sequencing. Therefore, we used an LRT (see Methods) to confirm whether the observed difference between the genus-level abundance estimation of the 16S rRNA gene fragments and full-length 16S rRNA sequences is significant or not. After correcting for multiple testing, 15/18 results showed that the difference were significant (FDR <0.05, with an average FDR of 0.00076). For samples TS1, TS8 and TS9, the test gives FDR values of 0.06, 0.05 and 0.16, respectively, which are not significant at the level of 0.05. However, the overall results have showed there were potential biases during data generation that resulted in the difference between the abundance estimation of the 16S rRNA gene fragments and full-length 16S rRNA gene sequences for this gut microbiome data. After comparing the sequencing protocols of the two data, we tend to believe that the most likely source of bias occurred during priming. Thus, the observed difference between the two data may reflect the effect of primer bias in targeted sequencing on abundance estimation. These results are also consistent with the original study where the significant difference among the full-length, V2, V6 and 16S rRNA gene fragment data was reported [2].

It was also claimed in the original survey that the full-length and V6 data tended to show a depletion of Bacteroidetes, while the V2 and 16S rRNA gene fragment data tended to show a relative depletion of Firmicutes when compared with the full-length and V6 data. In order to prove this more rigorously, we used the Wilcoxon signed-rank test to test the significance (*p*-value <0.05) of the difference between the abundance estimation of each genus in the 16S rRNA gene fragments, full-length, V2 and V6 data. The results suggested that the abundance estimation of the 13 genera supported by the 16S rRNA gene fragment data was significantly different from the other three data types. Among them, three genera belonged to the phylum Bacteroidetes. Nine genera belonged to the phylum Firmicutes. We also found one genus that belonged to the phylum Actinobacteria.

The above analysis suggests that the abundance estimation using the 16S rRNA gene fragments would be significantly different from that of the other data types based on technical bias, possibly primer bias. For the human gut microbiome, the difference mainly resides in the phylum Bacteroidetes in which the abundance estimated from the 16S rRNA gene fragments is higher and the phylum Firmicutes in which the abundance estimated from the 16S rRNA gene fragments is lower. These results suggest that primer bias could result in highly skewed abundance estimation thus lending support to the usage of 16S rRNA gene fragment data. We may also conclude that results of previous targeted 16S rRNA sequencing-based surveys should be treated cautiously.

Furthermore, for validation purposes, we used 16S rRNA gene fragment data to reproduce another previous conclusion reached by using only the V6 data in the original publication [2], indicating that the diversity of the gut microbiome in the lean population is higher than that in the obese population (see Methods). The result is shown in Figure 5 and suggests that even if abundance estimation is highly biased, the overall high-level conclusions reached using targeted 16S rRNA sequencing data may still hold true due to the higher robustness of β-diversity analysis [17].

## Discussion

16S rRNA gene fragments did not gain popularity in the past mainly because of the availability issue (low coverage). However, the recent data explosion has finally made 16S rRNA gene fragments a topic worth investigating. The protocol of shotgun sequencing provides 16S rRNA gene fragment data natural immunity against the bias raised during priming and thus the potential of uncovering the true structure of microbial community. Our established analysis framework could serve as a good starting point for experimental design and making the comparison between 16S rRNA gene fragment-based and targeted 16S rRNA sequencing-based surveys possible. With further development of computational methods [12], [13] and databases [14], [15], [16], we believe this new data type may improve our understanding about the diversity of various microorganism communities.

An important direction for future studies is benchmarking by repeating some previous studies using 16S rRNA gene fragment data for potential difference in conclusions. Although we have shown that some high-level conclusions from targeted sequencing-based surveys may hold true, there is no guarantee such conclusions would never be affected by bias in abundance estimation.
